# Elderly Fallers Enhance Dynamic Stability Through Anticipatory Postural Adjustments during a Choice Stepping Reaction Time

**DOI:** 10.3389/fnhum.2016.00613

**Published:** 2016-11-29

**Authors:** Romain Tisserand, Thomas Robert, Pascal Chabaud, Marc Bonnefoy, Laurence Chèze

**Affiliations:** ^1^IFSTTAR, UMR_T9406, Laboratoire de Biomécanique et Mécanique des Chocs (LBMC), Université de Lyon, Université Claude Bernard Lyon 1Lyon, France; ^2^Laboratoire Interuniversitaire de Biologie de la Motricité (LIBM), Université de Lyon, Université Claude Bernard Lyon 1Villeurbanne, France; ^3^Service de Médecine Gériatrique, Centre Hospitalier Lyon SudPierre-Bénite, France

**Keywords:** balance, fall, elderly, anticipatory postural adjustments, dynamic stability, step initiation

## Abstract

In the case of disequilibrium, the capacity to step quickly is critical to avoid falling in elderly. This capacity can be simply assessed through the choice stepping reaction time test (CSRT), where elderly fallers (F) take longer to step than elderly non-fallers (NF). However, the reasons why elderly F elongate their stepping time remain unclear. The purpose of this study is to assess the characteristics of anticipated postural adjustments (APA) that elderly F develop in a stepping context and their consequences on the dynamic stability. Forty-four community-dwelling elderly subjects (20 F and 24 NF) performed a CSRT where kinematics and ground reaction forces were collected. Variables were analyzed using two-way repeated measures ANOVAs. Results for F compared to NF showed that stepping time is elongated, due to a longer APA phase. During APA, they seem to use two distinct balance strategies, depending on the axis: in the anteroposterior direction, we measured a smaller backward movement and slower peak velocity of the center of pressure (CoP); in the mediolateral direction, the CoP movement was similar in amplitude and peak velocity between groups but lasted longer. The biomechanical consequence of both strategies was an increased margin of stability (MoS) at foot-off, in the respective direction. By elongating their APA, elderly F use a safer balance strategy that prioritizes dynamic stability conditions instead of the objective of the task. Such a choice in balance strategy probably comes from muscular limitations and/or a higher fear of falling and paradoxically indicates an increased risk of fall.

## Introduction

Falling is a common and unexpected event that is a concerning health problem for the elderly population (World Health Organisation, 2008). Normal aging increases the risk of fall (Rubenstein, 2006), because of a reduced capacity to use the different resources involved in the control of balance (Horak, 2006). The physical consequences of a fall are more severe than for a young person (van Dieën and Pijnappels, 2008) and falls induce psychological issues, notably by increasing the fear of falling (FoF; Maki et al., 1991). As such, falls currently represent a large and increasing health cost for societies (Stevens et al., 2006; World Health Organisation, 2008). Early identification of community-dwelling elderly that are at risk of fall is a priority, in order to: (1) prevent them from the loss of different capacities leading to dependency and frailty; and (2) reduce the health costs of falls.

In community-dwelling elderly, “most falls occur as a result of an inability to react appropriately [to the imbalance] and produce an effective compensatory response” (Brauer et al., 2002). A natural, effective and privileged reaction to recover when balance is compromised is taking a step (Rogers et al., 1996; Maki and McIlroy, 1997). The choice stepping reaction time test (CSRT; Lord and Fitzpatrick, 2001) is a simple test to assess the capacity of a person to rapidly trigger and execute a step. The subject has to step as quickly as possible on one of several targets placed in front or around her/him. The time to reach the targets is an effective way to assess the risk of fall in elderly, as several studies showed that elderly fallers (F) have significantly longer performances compared to non-fallers (NF; Lord and Fitzpatrick, 2001; Melzer et al., 2007; St George et al., 2007; Ejupi et al., 2014). Moreover, the time to perform the CSRT appears to be a good predictor for the future risk of fall (Pijnappels et al., 2010). However, the reasons why the CSRT predicts this risk are not well established. In particular, it has been shown in simple (one leg, one target) stepping reaction time (RT) condition that elderly F are able to move their foot as fast as NF (White et al., 2002; Melzer et al., 2007). So the difference is probably made before, i.e., during the mechanisms that precede the step.

A voluntary step initiation is a self-perturbation of balance, with a modification of the base of support (BoS) and a transition from a static to a dynamic situation. To keep balance, coordinated muscular activations preceding the voluntary focal movement, namely anticipatory postural adjustments (APA), are performed (for a review see Bouisset and Do, 2008). They are part of the motor command elaborated by the central nervous system (CNS; Massion, 1992; Aruin and Latash, 1995; Brunt et al., 1999, 2005). In step (or gait) initiation, their functional role is to put the whole-body center of mass (*CoM_WB_*) in motion: (1) in the desired direction; and (2) toward the future stance foot (Winter, 1995). This strategy reduces the subject’s mediolateral instability during the forthcoming single support phase (Jian et al., 1993; Patla et al., 1993; Lyon and Day, 1997), where the BoS is reduced to only one foot. The motor program of this strategy has been well described, with coordinated ankle and hip muscles activations and inhibitions (Crenna and Frigo, 1991; Brunt et al., 1999). This coordination creates joint torques that move the center of pressure (CoP) backward and laterally (Brenière et al., 1987; Jian et al., 1993; Winter, 1995; Lyon and Day, 1997). Then, the movement of the subject’s *CoM*_WB_ is principally driven by gravity effects during the swing phase (SP; Lepers and Brenière, 1995; Lyon and Day, 1997).

If APA are a very automatized postural control process, they are not invariant. They are adapted by the CNS to the external context, depending on the own resources of the subject (Patla et al., 1993; McIlroy and Maki, 1999; Luchies et al., 2002; Zettel et al., 2002; Yiou et al., 2012). In the context of a simple step initiation without a specific target, the studies that were interested in step preparation phases showed that elderly have APA elongated in time and reduced in amplitude compared to young adults (Halliday et al., 1998; Polcyn et al., 1998; Luchies et al., 2002). In the context of a CSRT, similar results have been found for elderly compared to young (Patla et al., 1993; Luchies et al., 2002) and for elderly F compared to NF, under normal (Lord and Fitzpatrick, 2001; St George et al., 2007) and dual-task conditions (Melzer et al., 2007; St George et al., 2007; Uemura et al., 2012a). Moreover, liftoff time is increased in CSRT compared to a simple RT test, increasing the landing time of the stepping foot (Luchies et al., 2002). So, the adaptable APA phase seems to be the major reason why the landing step timing is increased in elderly, and particularly in F, during a CSRT.

Why are APA elongated in time in elderly F? First, it is reported in the literature that a high FoF is associated to APA elongated in time and reduced in amplitude (Maki et al., 1991; Adkin et al., 2000; Yiou et al., 2011; Uemura et al., 2012b) and elderly F have an increased FoF compared to NF (Lajoie and Gallagher, 2004). The FoF has been shown to reduce the attentional resources available (Gage et al., 2003) and movement reinvestment (Huffman et al., 2009). So, elderly F probably have reduced attentional resources available. Moreover, normal aging reduces cognitive capacities. A reduced cognitive capacity is correlated to a longer stepping performance in elderly F during the CSRT (Lord and Fitzpatrick, 2001; Pijnappels et al., 2010; Schoene et al., 2015). The APA phase is also lengthened in elderly F during the CSRT, under dual-task paradigm (Melzer et al., 2007; St George et al., 2007; Sturnieks et al., 2008). This is probably because they need more attentional resources than NF during postural tasks under dual-task (Brauer et al., 2002; Woollacott and Shumway-Cook, 2002). Finally, elongated stepping performance is related to reduced proprioception (Pijnappels et al., 2010) and both sensorial and muscular capacity (Lord and Fitzpatrick, 2001). The muscular capacity of the lower limb is affected in elderly F, particularly around the hips (Johnson et al., 2004; Inacio et al., 2014; Morcelli et al., 2016).

Few studies have focused on the mechanics of the APA and its consequences on the stability, in a population of elderly F during a CSRT. We only found three studies talking about stability in the interpretation of their results in the conditions of step initiation. Patla et al. (1993) showed that elderly have a longer weight transfer time than young adults during CSRT, which resulted in a slower stepping response. Notably, in case of lateral steps, they found that elderly need more time because they choose to load their swing leg first, which is a sub-optimal strategy. They interpreted it as a “safer” strategy that helps elderly to increase their balance conditions. Later, Luchies et al. (2002) observed a slower weight transfer and a larger percentage of weight on the stance foot for elderly compared to young adults, in both simple step initiation and CSRT. They also used the term “safer” to describe the stepping strategy used by elderly. Unfortunately, the population of these two studies did not include elderly F. In the context of an induced step under dual-task condition the elderly—and even more for those who experienced a fall—reduce their secondary task performance (Brauer et al., 2002). They would do so to focus most of the available resources on the postural control, and by extension to increase the stability. According to their results there could be a prioritization of a more “stable” balance strategy in elderly and particularly in elderly F. This would be observed because their CNS has better integrated than NF that falling engages the physical integrity. Nevertheless, there still is a lack in the literature of a precise biomechanical analysis of the dynamic stability for a group of elderly F during a CSRT.

To sum-up, elderly F are slower to step than NF under both normal and dual-task conditions of CSRT. As already observed in stepping tasks, a hypothesis would be that it comes from a lengthened APA phase, in an attempt to maximize their stability. The aim of this study is to investigate the characteristics of the APA for both F and NF community-dwelling elderly subjects, in normal CSRT conditions (i.e., without a secondary task). We expect that APA will be longer for F compared to NF, as a result of a strategy that elderly F use to increase their conditions for dynamic stability.

## Materials and Methods

### Population

Forty-four healthy subjects participated in this study. They were divided in two groups: elderly F and elderly NF. Subjects were retrospectively categorized as F if they experienced at least a fall in the past year. A fall was defined as “an event, following an imbalance, which results in a person coming to rest inadvertently to a lower level, involving an impact, consecutive to the balance recovery actions failure and not a result of a major intrinsic event or overwhelming hazard”. This definition was chosen based on previous literature (Tinetti et al., 1988; Hauer et al., 2006; Segev-Jacubovski et al., 2011). Headcounts and anthropometrical data of the two groups are summarized in Table 1.

**Table 1:** **Mean (standard deviation) anthropometrical and MMSE data relative to the participants**.

	Elderly F	Elderly NF
Number of subjects	20	24
Number of women	15	14
Right-shooters	16	20
Age *[years]*	76.0 (3.9)	74.2 (3.9)
Age range *[years]*	70–82	70–82
Height *[m]*	1.61 (0.10)	1.64 (0.09)
Weight *[kg]*	68.6 (12.2)	65.3 (11.9)
BMI *[kg.m^-2^]*	26.5 (3.7)	24.2 (3.5)
MMSE *[score]*	28.7 (1.4)	28.9 (1.0)

*No statistical difference (using a *T*-test) was seen between the two groups. BMI, Body Mass Index; MMSE, Mini Mental State Examination*.

All subjects were included if they: (1) were aged 70 or more; (2) performed at least 25 on the Mini Mental State Examination (MMSE); and (3) had no neurological, musculoskeletal or sensorial (vision and cutaneous sensation) disorders, after a medical inspection. Forty-four healthy elderly adults participated in the study. Their mean age, mass and height were 75 years (ranging from 70 to 82), 66 kg (45 to 95) and 1.62 m (1.50 to 1.95), respectively. All subjects provided written informed consent to the experiment as conformed to the Declaration of Helsinki and was approved by the ethics committee Comité de Protection des Personnes Lyon Sud Est III.

### Protocol

**Figure 1:**
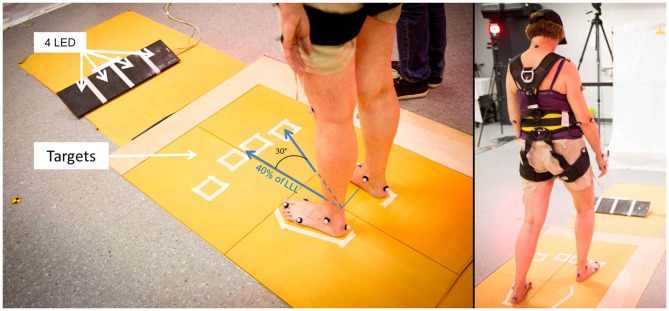
**Experimental set up for the choice stepping reaction time test (CSRT)**. Initial position of the subject, targets and board with light-emitting diode (LED) are shown on the left. Distance from the middle of the ankles and center of each target was 40% of the subject’s lower limb length (LLL). On the right, the same subject in the final position, after the lightning of the “Lateral-Right” target.

Each subject performed a CSRT. Subjects initially stood quietly, in a comfortable position, with arms along the body, eyes open and feet on two force platforms (60 cm × 40 cm, Bertec^®^, OH, USA). The positions of the feet was freely chosen by the subject and marked on the ground in order to repeat trials from the same initial posture. Four large targets (squared panels, 10 cm × 10 cm) were positioned on the ground at 40% of the subject’s lower limb length (LLL; see Figure 1). This distance was comfortable for the subject. The LLL was measured vertically, between the femoral trochanter center (Van Sint Jan, 2007) and the ground. Two targets were placed strictly anterior to the right and left foot (Central). The two others were placed 30° on each lateral side (Lateral). A light-emitting diode (LED) was placed in front of each target. LEDs were initially turned off. Instructions given to the subjects were: “as soon as one of the LED gets illuminated, step with the ipsilateral foot (i.e., left foot for the two left targets, right foot for the two right targets) on the corresponding target, as quickly as possible”. Each subject performed four trials on each target, randomly presented. To enhance the unpredictability of the imperative signal, the duration between the subjects said he/she was “ready” and the illumination of the LED was randomly chosen between 1 and 10 s.

Subjects were equipped with 39 reflective markers located on anatomical landmarks (Van Sint Jan, 2007) and recorded by eight cameras (Eagle 1.3 Mpx, Motion Analysis^®^, Santa Rosa, CA, USA) at 100 Hz sample frequency. Markers trajectories were filtered at 6 Hz with a Butterworth filter. The whole-body center of mass (*CoM_WB_*) trajectory was calculated using these markers trajectories and a segmental method (Dumas et al., 2007, 2015). Ground reaction forces (GRFs) were recorded at a sampling frequency of 1000 Hz with four force platforms, to integrate both the starting and landing areas (see Figure 1). The CoP was then estimated from the GRF measured by the force platforms at the same frequency. The CoP was estimated only when the resultant vertical force was higher than a threshold fixed at 20 N. No additional filtering was performed.

### Data Analysis

#### Step Phases Duration

**Figure 2:**
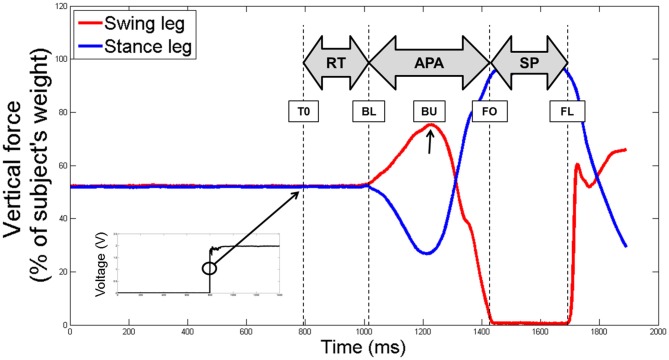
**An example of the raw vertical component of the ground reaction forces (GRFs) under the swing (in red) and stance (in blue) legs of a non-faller subject, before being time shifted.** The four particular instants identified (T0, BL, FO and FL) are reported with dotted lines. The beginning of the unloading is also reported with a black arrow (BU). The black squared signal in the bottom left shows the voltage signal of the LED, used to determine the T0. Abbreviations used: T0, first instant lighting the LED; BL, beginning of loading; BU, beginning of unloading; FO, foot off; FL, foot landing; RT, reaction time phase; APA, anticipated postural adjustments; SP, swing phase.

All signals (markers’ positions, GRFs and LEDs’ voltage) were recorded on the same data acquisition card (National Instruments USB 6218) and synchronized. They were further time shifted so that the beginning of the trial (T0) corresponded to the LED’s lightning (given by a raise in the LED’s voltage). Three particular instants were then defined relative to T0, based on the vertical components of the GRFs (see Figure 2):

- *Beginning of loading* (BL) which corresponds to the beginning of APA is the instant where the force under the swing leg increases more than two standard deviations of a reference period calculated between the beginning of the recording and T0;
- *Foot-Off* (FO) is the first instant where the swing leg force is inferior to 2.5% of the subject’s body weight;
- *Foot Landing* (FL) is the first instant where the swing leg force is superior to 2.5% of the subject’s body weight after FO.

Then, the three temporal phases were identified: the RT between T0 and BL, the anticipated postural adjustments (APA) between BL and FO and the SP between FO and FL.

#### APA and Swing Phases Analysis

**Figure 3:**
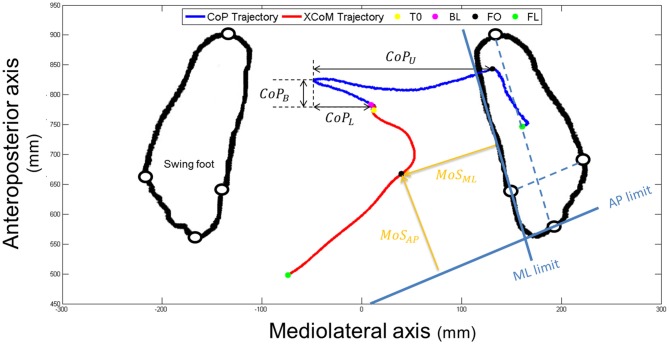
**An example of the horizontal center of pressure (CoP; in dark blue) and XCoM (in red) trajectories of a non-faller subject during the CSRT, seen from the top.** Particular instants identified (T0, BL, FO and FL) are reported with colored points on CoP trajectory. Only FO and FL are reported on both trajectories. Foot markers are represented with white disks circled in black. Amplitudes of the CoP displacements are represented with black double arrows (*CoP_B_* = backward; *CoP*_L_ = loading and *CoP*_U_ = unloading). The horizontal foot orientation is represented with dotted light blue lines that are then translated to the edges of the stance foot (solid light blue lines). Margin of stability (MoS) sizes at FO are represented with orange arrows. AP, anteroposterior; ML, mediolateral.

Specific variables were extracted and analyzed during the APA and the SP phases. First, we measured the presence of an APA error. An APA error was considered when the lateral trajectory of the CoP first moved toward the stance foot side—instead of the swing foot side—more than two standard deviations of the reference period measured between 0 and T0. Then, we were interested in the two subphases of APA used during forward step initiation (see Figure 3): a “loading” subphase where the CoP moves backward and toward the swing foot, leading the *CoM_WB_* to be put in motion forward and toward the stance foot; and an “unloading” subphase, during which the swing foot is unloaded, leading the CoP to move laterally under the stance foot (Jian et al., 1993). The beginning of the unloading subphase (BU)—corresponding the end of the loading subphase—was identified as the time when the vertical force under the swing leg was maximal (see Figure 2). The unloading subphase ended with the APA at FO. During the two APA subphases, the CoP displacements were characterized using the six following variables:

- *CoP_B_*: the maximal excursion of the CoP backward along the AP axis during the loading subphase;
- *CoP_L_*: the maximal excursion of the CoP along the ML axis toward the swing foot during the loading subphase;
- *CoP_U_*: the amplitude of the CoP displacement along the ML axis during the unloading subphase;
- *VCoP_B_*: the peak of the AP component of the velocity of the CoP during the loading subphase;
- *VCoP_L_* : the peak of the ML component of the velocity of the CoP during the loading subphase;
- *CoM_U_* : the peak of the ML component of the velocity of the CoP during the unloading subphase;

The CoP velocity was obtained by the first time derivative of the CoP trajectory, with a 2nd order lowpass digital Butterworth filter and a cutoff frequency of 20 Hz. Finally, during the SP, we analyzed the horizontal tangential velocity of the swing foot, using the first derivative of the ankle center trajectory given by the middle of the two malleolus markers. The horizontal distance traveled by the *CoM_WB_* between T0 and FL was also calculated.

#### Dynamic Stability: XCoM and MoS Analysis

The position of the XCoM in the horizontal plane was computed with the following equation (Hof et al., 2005):

(1) $$XCoM\;=\;\left(CoM_{WB}+\frac{1}{\omega_{0}}Co{\dot{M}}_{WB}\right)\cdot e_{proj}\;\omega_{0}\;=\;\sqrt{\frac{g}{h}}$$

*Co$\dot{M}$_WB_* is the vector of the *CoM*_WB_’s velocity, obtained by numerical derivation and filtering. *g* is the gravitational constant and *h* the distance along the vertical axis between the ankle and *CoM*_WB_’s position in static initial posture. The vector *e_proj_* projects the results in the horizontal plane of the laboratory coordinate system.

The dynamic stability was quantified at FO using the minimal distance between the positions of the XCoM and the edges of the stance foot, along both the AP and ML directions of the stance foot (see Figure 3). These variables, further referred as *MoS_AP_* and *MoS*_ML_, could be seen as the margin of stability (MoS; Hof et al., 2005) in these two directions. AP and ML directions of the stance foot were first defined as the lines passing through the markers positioned on calcaneus and 3rd toe and through the markers positioned on 1st and 5th metatarsal heads, respectively (dotted blue lines on Figure 3). The advantage of this method is that it takes into account the horizontal orientation of the foot. The anterior and medial edges of the BoS were then obtained by translating these lines to pass through the 1st metatarsal head marker and the 3rd toe marker, respectively (solid blue lines in Figure 3). Note that recent articles suggested the use of a functional BoS, i.e., a proportion of the initial BoS, instead of the mechanical BoS to correctly analyze the MoS values (Vallée et al., 2015; Hof and Curtze, 2016). However, the correct proportions to be used are still debated and using the mechanical or functional BoS will not change the meaning and interpretation of our results. At FO the BoS is the stance foot. *MoS*_AP_ and *MoS*_ML_ were calculated as the perpendicular distances between XCoM and the BoS edges (see Figure 3) and normalized by the BoS length (distance between the calcaneus and 3rd toe markers) and width (distance between the 1st and 5th metatarsal markers of the stance foot), respectively. For interpretation, the higher (and positive) these values, the higher the stability. Note that the XCoM being most of the time medial to the ML BoS edge (as it is shown on Figure 3), *MoS*_ML_ is quasi-systematically negative. It means that the subject is in condition of instability and, not surprisingly, that a static stable standing posture can only be reached by placing the swing foot laterally to the stance foot.

### Statistics and Graphic Representations

The steps on the left side were reflected about the laboratory AP axis to the steps on the right side. *T*-tests performed on the total duration comparing left and right target for both Central and Lateral conditions inside each group revealed probabilities to be different superior to 0.50 (for example in NF, *p* = 0.85 for Central and *p* = 0.64 for Lateral). So, right and left trials were combined in the two targets: Central and Lateral.

A first analysis on the frequency of APA error was performed with a χ^2^ test. Next, the normality of the distribution in the other variables was evaluated with a Shapiro-Wilk test. All of them were reported normal, so we tested them with two-way repeated measures ANOVA. The factors tested are the independent factor “Group” (F or NF) and the repeated factor “Target” (Central or Lateral). When an interaction was found, *post hoc T*-tests with the Bonferroni correction were performed. We did all statistical tests using the R^®^ software and a *p* < 0.05 was considered for a statistical difference.

For clarity, we choose to represent on the graphs the results for the two groups on each target even if an interaction was absent. In this case only the main factor effects of the ANOVA are reported. If an interaction was present, the results of the *post hoc* test are added to the main factor results coming from the ANOVA.

## Results

Seven hundred and four trials were collected. Twenty-Seven were instantly removed for the following reasons: subjects stepped with the wrong foot (19) or problems with forceplates data recordings appeared (signal partly or totally absent, 8). The 677 trials left were analyzed to detect the presence of APA errors. APA errors were observed in 21.6% (146) of the trials. Results of APA errors were 22.7% and 20.6% for F and 24.9% and 21.4% for NF in Central and Lateral targets, respectively. For both targets, there were no statistical difference between F and NF: χ^2^ = 0.02, *p* = 0.89 and χ^2^ = 0.03, *p* = 0.87 for Central and Lateral, respectively.

As no difference was seen between F and NF, we chose to analyze only the 78.4% left of the collected trials. So, the following results concern only the 531 “correct” trials of the initial 677.

In those trials, the results of the ANOVA tests have been summed up in Table 2.

**Table 2:** **Recapitulation of the results using two-way repeated measures ANOVA performed for all the variables in this study**.

		Group		Target		Interaction	
		F	p	F	p	F	p
Durations
	Total	9.86	**<0.01**	105.39	**<0.001**	<0.01	0.97
	Reaction time	2.34	0.13	0.25	0.62	2.01	0.16
	APA phase	13.01	**<0.001**	102.04	**<0.001**	0.10	0.75
	Swing phase	2.79	0.10	20.71	**<0.001**	0.49	0.49
	Loading subphase	5.98	**0.02**	54.80	**<0.001**	1.73	0.19
	Unloading subphase	6.84	**0.01**	34.59	**<0.001**	0.06	0.81
CoP amplitude
	*CoP_B_*	4.88	**0.03**	1.81	0.18	0.24	0.63
	*CoP_L_*	0.49	0.49	37.57	**<0.001**	0.04	0.83
	*CoP_U_*	1.60	0.31	26.84	**<0.001**	2.29	0.14
CoP velocity
	*VCoP_B_*	11.31	**0.02**	0.38	0.54	0.02	0.88
	*VCoP_L_*	0.94	0.34	8.37	**<0.01**	0.44	0.51
	*VCoP_U_*	2.17	0.15	3.52	0.07	2.80	0.10
MoS sizes
	*MoS_AP_*	4.64	**0.04**	7.93	**<0.01**	0.48	0.49
	*MoS_ML_*	2.45	0.13	385.11	**<0.001**	5.48	**0.02**
Swing phase
	Foot velocity	2.21	0.15	24.00	**<0.001**	0.01	0.94
*CoM_WB_* displacement		0.31	0.58	75.09	**<0.001**	0.53	0.47

*F and p from the ANOVA are provided. p inferior to 0.05 are indicated in bold*.

**Figure 4:**
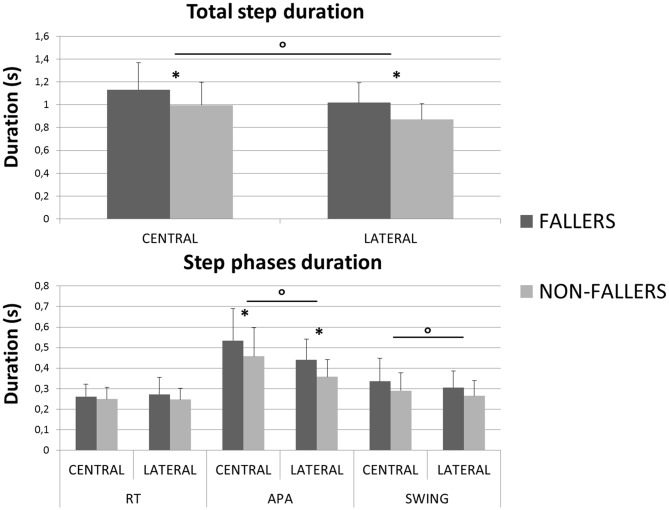
**Mean durations (with standard deviations) measured for both groups and targets during the CSRT.** On top are presented results for the total step duration. On the bottom are presented results for the three steps phases (RT, APA and Swing). *Indicates a significant effect of the main factor “Group”. °Indicates a significant effect of the main factor “Target”.

**Figure 5:**
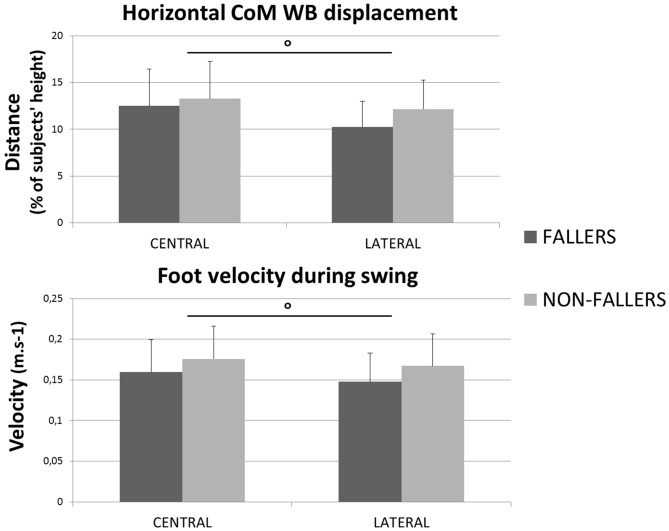
**Mean values (with standard deviations) measured of the *CoM*_WB_ displacement (top) and the velocity of the swing foot (bottom) for both groups and targets.** °Indicates a significant effect of the main factor “Target”.

A significant effect of the factor “Group” was found on the total step duration. F compared to NF needed 1131 ± 231 ms vs. 997 ± 175 ms in Central and 1019 ± 161 ms vs. 870 ± 117 ms in Lateral, to execute a quick step during the CSRT (see Figure 4, top). This observation was independent from the target, although no significant differences between F and NF were observed on the *CoM_WB_* displacement (see Figure 5, top). Indeed, for F compared to NF, the *CoM*_WB_ horizontal displacement was 12.5 ± 4.0% vs. 13.3 ± 4.0% of the subject’s height for Central and 10.2 ± 2.6% vs. 12.1 ± 3.0% of the subject’s height for Lateral. We also found an effect of the factor “Target”, indicating that the total step duration was significantly increased in Central compared to Lateral targets.

The analysis of each step phase duration (Figure 4, bottom) showed that APA was the only phase significantly elongated in elderly F compared to NF (534 ± 150 ms vs. 457 ± 139 ms in Central and 441 ± 98 ms vs. 357 ± 84 ms and in Lateral). As for the total step duration, this result was independent from the target and the mean values measured on Central targets were significantly higher than on Lateral targets.

The mean values measured on the RT phase duration for F compared to NF were 261 ± 61 ms vs. 272 ± 82 ms in Central and 249 ± 57 ms vs. 247 ± 55 ms in Lateral. Analysis of this phase reported neither effect of the factors “Group” nor “Target” (see Table 2).

For the SP duration and swing foot velocity, a significant effect of the factor “Target” was found, whereas the effect of the factor “Group” revealed trends (see Table 2, Figures 4, 5). Those trends indicated that the mean values for SP duration are always longer for F compared to NF (336 ± 111 vs. 290 ± 87 ms in Central and 305 ± 79 vs. 265 ± 73 ms in Lateral), and that the mean values for swing foot velocity were always smaller in F than in NF (0.16 ± 0.04 vs. 0.18 ± 0.04 m.s^−1^ in Central and 0.15 ± 0.4 vs. 0.17 ± 0.4 m.s^−1^ in Lateral).

**Figure 6:**
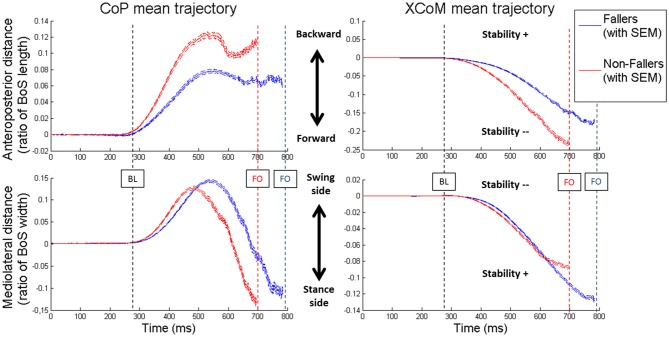
**Mean values of the CoP (on the left) and XCoM (on the right) trajectories from T0 to FO in Lateral targets.** For both groups the two components of the movement are presented: anteroposterior (on top) and mediolateral (on the bottom). Blue lines are the results for Fallers (F) and red lines for Non-fallers (NF). The lines represent the mean value and the standard error to the mean (SEM). For clarity, the BL is the mean of the two groups. Note that for the anteroposterior displacement of the XCoM (top right) the less negative the XCoM indicates a better stability while for the mediolateral displacement of the XCoM (bottom right) the less negative XCoM indicate a worst the stability. For this representation, the base of support (BoS) width has been calculated between the positions of the two 5th metatarsal head markers, along the mediolateral axis.

**Figure 7:**
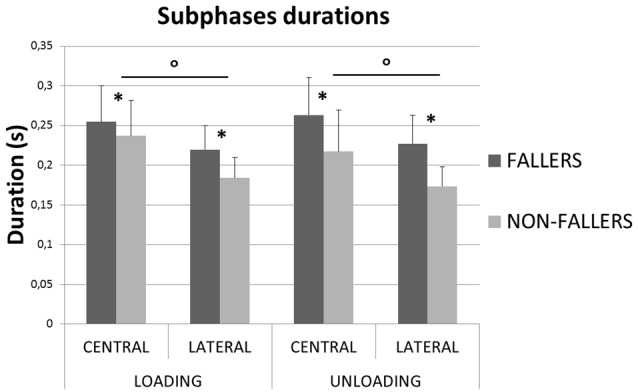
**Mean durations (with standard deviations) measured for the two subphases of APA for both groups and targets.** *Indicates a significant effect of the main factor “Group”. °Indicates a significant effect of the main factor “Target”.

In order to illustrate the APA mechanisms and their consequences on stability, the CoP and XCoM trajectories were plotted between T0 and FO. Results for Lateral targets are provided in Figure 6. Similar patterns were observed for Central targets. For clarity, all trajectories have been normalized on zero. Only for the representation, the BL instant has been averaged between the two groups. This figure highlights the differences in APA between F and NF and their consequences on the stability. First, as previously mentioned, we observed that the APA duration was elongated in F. Indeed, FO arose around 100 ms later in F than in NF. Also, the plot of the CoP displacement along the ML axis illustrates well the two APA subphases (bottom left in Figure 6): the “loading” is when the CoP move to the swing-foot side while the “unloading” is when the CoP moves to the stance-foot side. The duration of these two subphases (see Figure 7) were significantly increased for F compared to NF: 254 ± 45 ms vs. 237 ± 45 ms in Central and 220 ± 30 ms vs. 183 ± 26 ms in Lateral to complete the loading subphase; 263 ± 47 ms vs. 217 ± 52 ms in Central and 227 ± 35 ms vs. 173 ± 24 ms in Lateral to complete the unloading subphase.

**Figure 8:**
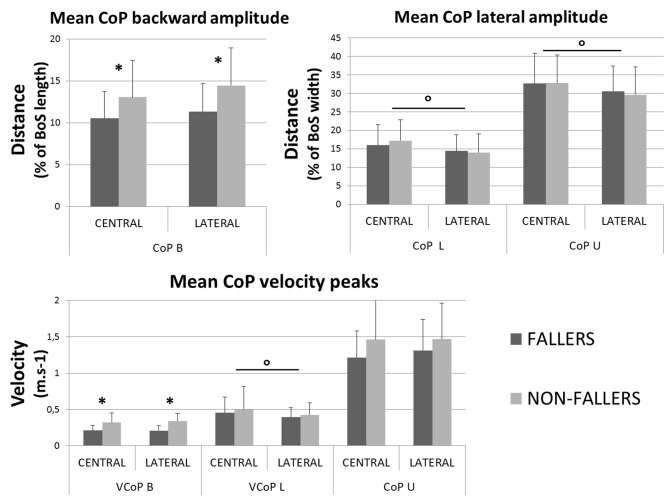
**Mean characteristics (with standard deviations) of CoP movement measured for both groups and targets during APA.** Backward (top left) and mediolateral (top right) maximal amplitudes are presented with velocity peaks (bottom). *Indicates a significant effect of the main factor “Group”. °Indicates a significant effect of the main factor “Target”. Note that here the BoS width refers to the initial BoS width, which is calculated between the positions of the two 5th metatarsal head markers, along the mediolateral axis.

**Figure 9:**
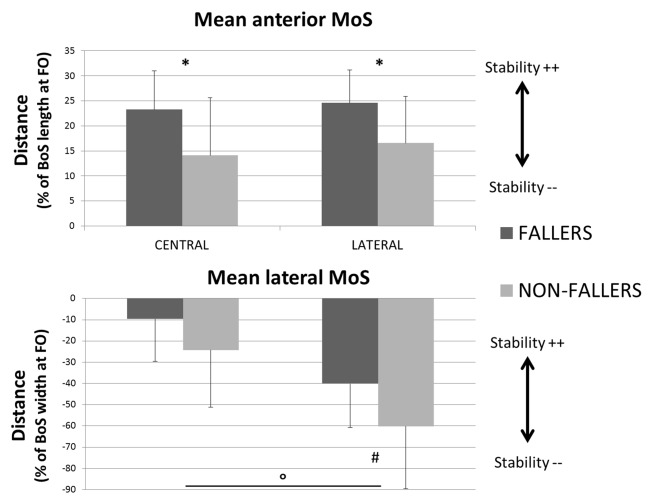
**Mean values (with standard deviations) of the MoS measured at FO for both the two groups and targets.** Anteroposterior component is presented on the top and mediolateral component on the bottom. Indicators of the quality of stability are provided for each of them (Stability ++ = a higher stability, Stability −− = a smaller stability). *Indicates a significant effect of the main factor “Group”. °Indicates a significant effect of the main factor “Target”. ^#^Indicates a significant difference between F and NF measured with the *post hoc* test. Note that here the BoS width is the width of the stance foot, the current BoS at FO.

Two different CoP displacement strategies were observed in the AP and ML directions, respectively (see Figure 6, left panels). Both resulted in a similar effect on the stability (see Figure 6, right panels): an increased stability in the AP direction and a less important instability (the XCoM is mostly external to the BoS at FO) in the ML direction, for the elderly F compared to NF (see Figures 8, 9).

- In the AP direction F moved their CoP less backward than NF: 9 ± 3.8% vs. 13.4 ± 5.5% of the BoS length in Central and 9.8 ± 3.9 vs. 14.7 ± 5.7% of the BoS length in Lateral. They also moved their CoP slower than NF: 0.21 ± 0.07 m.s^−1^ vs. 0.32 ± 0.12 m.s^−1^ in Central and 0.21 ± 0.07 m.s^−1^ vs. 0.34 ± 0.11 m.s^−1^ in Lateral. Consecutively, this strategy resulted in a smaller forward displacement of the XCoM (see Figure 6) and a significantly increased *MoS_AP_* (23.2 ± 7.7% vs. 14.1 ± 11.5% of the BoS length in Central and 24.6 ± 6.6% vs. 16.6 ± 9.2% in Lateral).
- In the ML direction there were no significant differences between F and NF in the amplitude of CoP displacements. For F compared to NF, we measured mean CoP displacements of 16.0 ± 5.6% vs. 17.2 ± 5.7% of the initial BoS width in Central and of 14.4 ± 4.4% vs. 14.0 ± 5.0% of the initial BoS width in Lateral during the loading subphase. During the unloading subphase, the amplitude of this displacement was 32.7 ± 8.1% vs. 32.8 ± 7.5% of the initial BoS width in Central and 30.6 ± 6.7% vs. 29.6 ± 7.4% in Lateral. We also did not found any significant differences between F and NF for the CoP velocity peaks (*VCoP_L_* and *VCoP*_U_). For F compared to NF, the mean *VCoP*_L_ measured were 0.46 ± 0.21 m.s^−1^ vs. 0.50 ± 0.31 m.s^−1^ in Central and 0.39 ± 0.13 m.s^−1^ vs. 0.42 ± 0.16 m.s^−1^ in Lateral. We found however a significant effect of the factor “Target” (*p* < 0.01), with mean values measured on Central targets significantly higher than those on Lateral. *VCoP*_U_ was 1.21 ± 0.37 m.s^−1^ vs. 1.45 ± 0.64 m.s^−1^ in Central and 1.31 ± 0.43 m.s^−1^ vs. 1.46 ± 0.50 m.s^−1^ in Lateral, for F compared to NF. Nonetheless, longer APA duration for F tended to induce a larger lateral displacement of the XCoM at FO (see Figure 6). Whether the *MoS*_ML_ was not significantly different between F and NF (–9.6 ± 19.8% vs. –24.2 ± 27.1% of the stance-foot BoS width in Central and –40.0 ± 20.6 vs. –60.0 ± 29.4% of the stance-foot BoS width in Lateral), the significant interaction Group * Target (see Table 2) showed that this result depended on the Target. Independent analysis of each target revealed that F had a significantly larger *MoS*_ML_ than NF only for the Lateral targets (*p* < 0.01 after Bonferroni correction).

## Discussion

### Step and Step Phases Durations

As previously in the literature (Lord and Fitzpatrick, 2001; St George et al., 2007), we found that elderly F need more time to perform a CSRT under normal conditions (i.e., no secondary task). This result confirms that this test is relevant to identify community-dwelling elderly that are at risk of fall, with a simple measurement (the total duration of the step) conceivable outside of the laboratory (clinical environment, home, etc.) (Lord and Fitzpatrick, 2001; Schoene et al., 2011; Ejupi et al., 2014). The total mean durations obtained in our study are shorter than in Lord and Fitzpatrick (2001) study: 1075 ms vs. 1322 ms for F and 933 ms vs. 1168 ms for NF. This difference could be explained by the fact that we removed the trials with APA errors from analysis. Interestingly, the mean difference between the two groups is similar in both studies (~150 ms). So, the total step duration difference between F and NF does not seem to be influenced by the presence of APA errors. Despite the fact that they need more time to step, elderly F made similar steps (see results in Figure 5) and as many APA errors as NF. This last result may seem contradictory with those from the previous studies (St George et al., 2007; Sparto et al., 2014) who found that subjects who make more APA errors are mostly the subjects with a history of fall and with a high risk of fall, respectively. It could be explained by the fact that Sparto et al. (2014) used purely lateral targets and reported an “error” when a loading subphase was observed, which is a very strict criterion (the presence of a loading subphase being more a sub-optimal response than an error). In St George et al. (2007) study subjects were under dual-task most of the time, which could have complicated the target identification for F. To sum-up, we found that elderly F are slower but able to execute the same step as NF during the CSRT and that the presence of an APA error is apparently not a reason to explain why they need more time to step during this test.

Regarding the step phases independently we found similar RT phase duration for F and NF. The mean value obtained for the RT is close to previous measurements in elderly (Luchies et al., 2002), but differs from the longer durations measured by Patla et al. (1993) (~400 ms vs. 280 ms in our study) and St George et al. (2007) in their condition without secondary task (~350 ms). In Patla et al. (1993), targets also involved posterior steps. As the CoP has to move first forward in posterior steps, subjects may have taken more time to ensure the identification of the direction of the target before starting APA. In St George et al. (2007) study, this difference could be explained by the determination of the beginning of APA: they took the first activation of gastrocnemius that, as soleus, are ankle plantar flexors who are firstly turned off during the forward step initiation (Crenna and Frigo, 1991). Moreover, our results indicate that the F and the NF have similar SP durations and swing foot velocity (see Figures 4, 5).

So, an important result of this study is that the total duration of the step is elongated in elderly F compared to NF because their APA phase is elongated. This result is similar to what Patla et al. (1993) found for elderly compared to young adults, and the timing difference between F and NF related here is similar to St George et al.’s (2007) measurements in their condition without secondary task. It confirms the hypothesis that the difference between F and NF is made during the mechanisms preceding the step execution. Our result is reinforced by the fact that APA of elderly F last longer than those of NF, independently of the direction of the target (see Figure 4). Indeed, even for the Lateral targets, a situation that needs *a priori* reduced APA because of the advantages of the gravity effects on the frontal plane during the SP (Patla et al., 1993; Lepers and Brenière, 1995; Lyon and Day, 1997; Sparto et al., 2014), this difference is highly significant (*p* < 0.001). So, as Patla et al. (1993) observed for elderly in lateral steps, elderly F may chose not to take advantage of the gravity as much as elderly NF do during the execution of APA for lateral steps.

### Two Different Balance Strategies, Depending on the Axis

Looking at the biomechanical mechanisms occurring during the APA, we showed that elderly F tend to keep their XCoM closer to the stance foot at FO than NF (see Figure 6). This situation allows them to increase their conditions for dynamic stability at this particular instant, i.e., when the BoS is reduced to only one foot (although this result was not significant in the ML direction for the Central targets). Moreover, as the body behaves almost as a passive mechanism during the SP (Lyon and Day, 1997) and as the swing characteristics observed here (foot location at FL and SP duration) are unchanged between F and NF, differences in dynamic stability at FL could be expected from the differences in XCoM locations at FO. In particular, the XCoM is further from the stance foot at FO for NF (see Figure 6). It likely induces a larger ML displacement of the XCoM during the SP and could result in a smaller dynamic stability at FL for NF compared to F, similar to what was observed at FO. This should nevertheless be confirmed by proper estimations of the XCoM and of the BoS at FL. Interestingly, the increased conditions for stability were obtained through two different strategies observed in the ML and AP directions.

In the ML direction, we did not find any statistical difference in the CoP trajectory between F and NF, both in amplitude and velocity variables (*CoP_L_*, *CoP*_U_, *VCoP*_L_ and *VCoP*_U_, respectively, see Figures 6, 8). Longer durations of both loading and unloading subphases (see Figure 7) implied however that the CoP stayed lateral to the *CoM*_WB_ on the swing foot side for a longer time in F than in NF. Consequently, the torque that propels the *CoM*_WB_ toward the stance leg is more efficient in F and so the XCoM is more shifted toward the stance foot (see Figure 6). Thus, the ML instability at FO is reduced: *MoS*_ML_ is less negative (although it was only significant for Lateral targets, see Figure 9). This elongated duration implies a poorer performance in the CSRT task (Patla et al., 1993; Lord and Fitzpatrick, 2001). Interestingly, a similar result in terms of stability could be obtained without lengthening the APA phase duration. It would consist in increasing the CoP peak velocity or excursion, i.e., in performing more efficient APA than NF. Why F do not to use this later strategy remains an open question. Two hypotheses could be proposed: (1) a physical limitation, in particular in the hip abductors/adductors that are primarily responsible for the CoP ML displacement (Winter, 1995); and (2) the FoF that would prevent the subjects to unbalance themselves more quickly. This study does not bring firm arguments for or against one of these hypotheses. By elongating their APA without modifying the amplitude, F subjects may have tried to minimize the muscular effort (Zettel et al., 2002). Indeed, a larger CoP displacement in the mediolateral direction (excursion and peak velocity) will require a high level of muscular strength at the hip abductors/adductors. It has been reported that elderly and particularly F have both weaker hip adductor/abductors capacity (Johnson et al., 2004; Inacio et al., 2014; Morcelli et al., 2016) and a reduced lateral stability (Rogers et al., 2001; Johnson-Hilliard et al., 2008). Elderly F also have a higher FoF (Maki et al., 1991; Vellas et al., 1997; Lajoie and Gallagher, 2004). A high FoF affects the development of APA (Adkin et al., 2000; Yiou et al., 2011), and so F subjects may have tried to reduce the risk to fall on a particular side. Finally, it could be a combination of these two hypotheses. Nevertheless, it is remarkable that in the present study F performed at least as well as NF in terms of CoP excursion and peak velocity in the ML direction. As such, a lengthened APA phase measured during a CSRT test appears to be an earlier indicator of the risk of fall for community-dwelling elderly subjects than the capacity to move the CoP during the APA.

The situation is different in the AP direction: elderly F limited the CoP backward excursion (*CoP_B_*) and peak velocity (*VCoP*_B_) compared to NF (see Figure 8). According to Brenière and collaborators model (Brenière et al., 1987; Lepers and Brenière, 1995), it means that during APA the F reduced the distance between the CoP and the *CoM*_WB_ in the AP direction. Consecutively, F did not create a forward propulsive torque as efficient as NF. This mechanism led to a smaller displacement of the XCoM in the forward direction and to an increased stability at FO. This is typically what we observed for F compared to NF (see Figures 6, 9). We could interpret these results in two different ways: (1) F cannot move their CoP further or faster backward, due to physical limitations or a higher FoF, and the APA last as long as the XCoM is forward enough to step; and (2) F chose to decrease the CoP excursion in order to enhance the stability at FO. In this case the decrease is even more pronounced that APA duration is increased, probably due to limitations in the ML direction (see paragraph above). Nonetheless, our results on elderly F show that they were as able as NF in: (1) moving their CoP in the ML direction inside the BoS; and (2) moving their foot during the SP (a part of the movement that also engages muscular capacity). Again, this study does not bring enough firm arguments pro or against any of these interpretations. According to our results the second interpretation seems however to correspond the best.

Different APA strategies in ML and AP directions are used by F compared to NF. Both resulted in an increase of the dynamic stability at FO. It seems that the increase in APA duration is primarily due to limitations of the ML direction. A lengthened APA phase measured during a CSRT appears to be an earlier indicator of the risk of fall than the capacity to move the CoP, in community-dwelling elderly subjects.

### Two Strategies that Aim to Increase Stability Instead of Rapidity in Elderly F

As discussed previously, elderly F displays a higher stability at FO. One of the counterpart is that they take less advantage of the disequilibrium torque given by gravity to propel the body in the direction of the targets, at the beginning of the SP (Lepers and Brenière, 1995; Lyon and Day, 1997). Another negative consequence is that it necessitates longer APA duration that decreases performances at the CSRT (Lord and Fitzpatrick, 2001).

To interpret those results, we can see the CSRT as a test involving two “tasks” for the CNS: stepping on the target as fast as possible (rapidity) and maintaining balance (stability). The results we observed in this study resemble to a “safer” strategy—as previously suggested by Patla et al. (1993), Brauer et al. (2002) and Luchies et al. (2002)—where the elderly F seem to enhance stability to the detriment of rapidity. In a different context, Brauer et al. (2002) showed that elderly having balance troubles prioritize stability instead of a dual task, probably because they involve maximal attentional resources in the accomplishment of the primary “task” (i.e., maintain balance). Similarly to what they suggested, a hypothesis would be that elderly F may see stability as the “primary task” during the CSRT and choose to prioritize it. We suggest that F make a choice because they “go against” the instructions of the test which were clearly to give priority to the rapidity.

This choice could also be qualified as a “conservative” strategy (Nakano et al., 2016), because elderly F seems to use unnecessary large conditions of stability at FO—as elderly do regarding to young adults in lateral steps (Patla et al., 1993). This inability of F to limit, in reasonable proportions, their stability at FO could even be seen as a limited capacity to adapt their motor command to the external context. Elderly F may perform this “conservative” strategy during the CSRT because the initiation of a voluntary step can always be delayed. In a more demanding context, such as a protective step (Rogers et al., 1996; Maki and McIlroy, 1997), this strategy would probably induce balance issues and a higher risk of fall. During protective steps the APA are usually shortened in time and reduced in amplitude in the ML direction to adapt to the perturbation (McIlroy and Maki, 1999) and the lateral balance has been shown to be the most determinant capacity for F to prevent from falling (Rogers et al., 2001; Johnson-Hilliard et al., 2008). The results pointing out that elderly F prioritize a more stable strategy than NF at FO could be interpreted as a poorer control of balance and an increased risk of fall. It has recently been suggested that an increased MoS is an indicator of a decreased control of lateral balance and a higher risk of fall during gait (Vistamehr et al., 2016).

Why would elderly F prioritize a more stable strategy than NF at FO? As the postural control is complex and involve multiple capacities and processes (Horak, 2006), there is never only one reason. If elderly F are effectively choosing a more stable balance strategy, it is probably because of the use of different processes and the integration of their own capacities, which are different from one subject to another. Reasons could be found in numerous capacities and processes, as the literature has already shown in the past (sensorial, cognitive, muscular, psychological). It appears important to us to point out that all subjects of our group of elderly F have one characteristic in common: they fell in the past year. This has probably significantly increased their FoF (Maki et al., 1991; Lajoie and Gallagher, 2004). Then, as the FoF reduces attentional resources available (Gage et al., 2003) and movement reinvestment (Huffman et al., 2009), an interaction between FoF and cognitive processes that acts in APA elaboration may have influenced their choice. A higher FoF is probably the most important reason why we observed this balance strategy in elderly F.

### Limitations

This study presents several limitations. The location of our “Lateral” targets may not have been enough lateral (see Figure 1). The main limitation for balance in elderly F seems to come from the ML control of the CoP and our results are the most significant for these targets. More pronounced effects may be obtained using, for instance, 45° targets rather than 30°.

Another limitation comes from the fact that there is no simple RT test in that study, such as in Luchies et al. (2002). Such data could have helped to determine if sensory processing was reduced in our group of F and/or if they needed more time than NF to program the correct APA during a CSRT.

We did not study our population under a secondary task during this test, making impossible to know if our F subjects suffered from reduced attentional or inhibition capacities. As the balance strategy observed may come from a choice of a more “safer” or stable strategy, it could have been interesting to see if FoF has an interaction with the decisional process. We cannot currently conclude on those processes, which need to be further investigated.

## Conclusion and Perspectives

The results presented here confirmed our hypothesis. Elderly F have an elongated performance in the CSRT due to longer APA phase. By lengthening the APA duration in the ML direction without increasing the CoP displacement performance (excursion and peak velocity), F increase the MoS at FO.

This strategy can be qualified as a “safer” strategy—as suggested previously by Patla et al. (1993), Brauer et al. (2002) and Luchies et al. (2002)—used to the detriment of the CSRT performance. This strategy probably comes from a choice due to a higher FoF, which changes the way posture and balance are controlled (Maki et al., 1991; Adkin et al., 2000; Brauer et al., 2002; Huffman et al., 2009; Yiou et al., 2011) and/or an attempt to minimize the muscular effort (Zettel et al., 2002). In a more demanding environment, this incapacity to adjust the stability to the task would probably induce balance issues and a higher risk of fall. Programs for the risk of fall prevention in community-dwelling elderly adults should focus on helping elderly F to get confidence back in their capacity to manage balance in different situations and, by so, improve balance performances.

In perspective of this study, we will look more specifically at the trials with APA errors. It would be indicative to know how elderly F correct these errors. Such information would particularly inform about their inhibition capacity, as Sparto et al. (2014) showed. Further improvements of this test are also to consider, like for example use of 45° targets during a CSRT.

## Author Contributions

RT contributed with project creation, data collection, data analysis and drafted the manuscript. TR contributed with project creation and data analysis. PC contributed in data analysis. MB contributed with project creation and recruitment of the subjects. LC contributed with project creation, data collection and data analysis. All authors discussed the results and participated in the revision of the manuscript.

## Funding

RT held a doctoral fellowship from La Région Rhône-Alpes.

## Conflict of Interest Statement

The authors declare that the research was conducted in the absence of any commercial or financial relationships that could be construed as a potential conflict of interest.
